# Dignity of Work and at Work: The Relationship between Workplace Dignity and Health among Latino Immigrants during the COVID-19 Pandemic

**DOI:** 10.3390/ijerph21070855

**Published:** 2024-06-29

**Authors:** Thespina J. Yamanis, Samhita Rao, Alexandra J. Reichert, Rachel Haws, Taryn Morrissey, Angela Suarez

**Affiliations:** 1 School of International Service, American University, 4400 Massachusetts Ave. NW, Washington, DC 20016, USA; sr21.research@gmail.com; 2 Department of Anthropology, Vanderbilt University, 2301 Vanderbilt Place, Nashville, TN 37235, USA; alexandra.reichert@vanderbilt.edu; 3 Department of International Health, Johns Hopkins Bloomberg School of Public Health, 615 N. Wolfe St., Baltimore, MD 21205, USA; rhaws@jhmi.edu; 4 School of Public Affairs, American University, 4400 Massachusetts Ave., Washington, DC 20016, USA; morrisse@american.edu; 5 La Clinica del Pueblo, Washington, DC 20009, USA; asuarez@lcdp.org

**Keywords:** social determinants of health, Latino, Latina, undocumented immigrants, health equity, noncitizens, epidemic, SARS-CoV2, dignity, work

## Abstract

Latino immigrants living in the United States were highly vulnerable to the health and economic consequences brought on by the COVID-19 pandemic. We use the conceptual framing of workplace dignity, worth that is acknowledged based on performance of job responsibilities, to explore Latino immigrants’ experiences during the early months of the pandemic. A qualitative study was conducted with *La Clínica del Pueblo* (*La Clínica*), a community health center serving low-income Latino immigrants. From June to December 2020, we conducted in-depth video interviews with 29 Latino immigrant clients to explore pandemic-related challenges, including workplace changes, discriminatory experiences, and effects on health. We conducted thematic analysis using Dedoose software. Nearly half of participants were undocumented immigrants. Most participants were unemployed or underemployed due to the pandemic and 26–49 years of age; one-third were still working, and one-quarter were 50 years or older. About half were cisgender women and two were transgender women. Employed participants experienced a lack of dignity through being socially isolated and stigmatized at work; receiving no compensation for their extra labor or for sick leave; and experiencing discriminatory labor practices. Unemployed participants experienced a lack of dignity in being the first to lose their jobs without government support; losing self-esteem; and not being rehired. Participants associated denial of dignity with worsening health conditions and increased anxiety and depression. Our study suggests that denial of workplace dignity—through job loss, underemployment, and poor working conditions—is linked to adverse health outcomes for Latino immigrants. More research should recognize workplace dignity as an important social determinant of health.

## 1. Introduction

The COVID-19 pandemic and its associated economic repercussions disproportionately affected the United States (U.S.) Latino population [1,2]. During the COVID-19 pandemic, Latinos living in the U.S. were more likely to experience what Perry and Aronson term “pandemic precarity”—material and financial insecurity attributable to the pandemic [3]. Overall, Latinos experienced 23% of initial job losses due to the pandemic, while constituting only 16% of the population [4]. A January 2021 Pew Research Center survey reported that 49% of Latino adults, 58% of Latino immigrants without a green card, and 53% of Latino immigrants with a green card reported that they or someone in their household suffered a job loss, layoff, or pay cut since the pandemic began, each higher than 44% of the overall population [5]. Job loss and changes likely resulted in negative physical and mental health effects for Latino immigrants. Nevertheless, descriptions of these effects and the pathways through which job losses and changes affected health for Latino immigrants have been underreported.

Recently, more attention has been placed on workplace conditions as a key social determinant of health [6,7]. Researchers have called for greater inquiry into the working conditions of international migrants, a large and growing population [6]. Furthermore, a recent review, as well as the U.S. National Institutes of Health, emphasized that more theoretical frameworks are needed that explain the processes through which workplace conditions are linked to health [8,9].

One pathway through which workplace conditions may affect health is workplace dignity, a concept theorized by Kristen Lucas as worth that is acknowledged based on performance of job responsibilities, as well as self-esteem and status derived from engaging in doing work itself [10]. Lucas argues that workplace dignity is theoretically distinct from dignity alone, defined as a personal sense of worth, respect, esteem, or value derived from one’s social position [10], which has long been used to defend human rights of vulnerable populations, including migrant workers [11]. Others have acknowledged that the workplace is a place where dignity can be both created and put at risk [12,13]. Nevertheless, the theoretical concept of workplace dignity has been underused in research on work as a social determinant of health. Lucas’ theory of workplace dignity privileges workers’ own perspectives on work and links dignity to workers’ social position, thus providing a useful framing for immigrant workers whose ability to work is often explicitly tied to the rights conferred by their legal immigration status.

**Figure 1:**
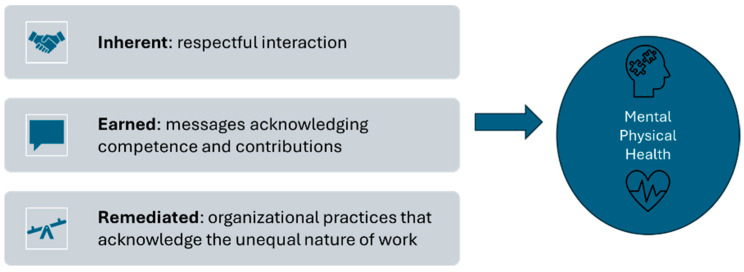
Description of three types of workplace dignity, with examples, as described by Lucas [10]. The presence of dignity can affect mental and physical health in positive ways, while the absence of dignity can affect mental and physical health in negative ways.

Lucas’ theory consists of three types of workplace dignity: inherent (or internal) dignity, earned dignity, and remediated dignity [10] (see Figure 1). Inherent dignity is the belief in an unconditional, intrinsic worth that all people have as human beings and is recognized through respectful interaction; conversely, rudeness can deny inherent dignity. Earned (or meritocratic) dignity is derived from contributions at work, affirmed through messages of competence and contribution and denied by public criticism or insult of job role or performance [10,13,14]. Remediated dignity restores dignity denied due to structural inequalities in the workplace or when a worker is treated as a replaceable tool to perform a job; remediated dignity is demonstrated through social interactions and organizational practices that reveal and acknowledge the instrumental and unequal nature of work [10]. Importantly, Lucas observes that although dignity is a positive concept, dignity is best understood in lived experience when it is *denied* rather than when it is affirmed.

Affirming or denying a worker’s dignity can thus theoretically affect health outcomes in positive and negative ways. Employment can create opportunities to recognize inherent dignity and receive esteem in the form of earned dignity. However, asymmetrical power dynamics in the workplace, to which immigrant populations are uniquely vulnerable, allow for dehumanization, strip workers of their dignity, reinforce instrumentality and inequality, and ultimately threaten workers’ mental and physical health. Job loss, underemployment, and unemployment effectively deny workers earned dignity of work and at work and can adversely affect mental health. Much workplace dignity scholarship to date has focused on vulnerable populations, who are typically economically and socially disadvantaged by societal or occupational inequalities. Nevertheless, little is known about the psychological and behavioral consequences of workplace dignity [15], particularly among immigrant populations.

### 1.1. Workplace Vulnerabilities for Latino Immigrants

Latino immigrants were likely denied dignity at work because they faced the COVID-19 pandemic with few personal or workplace protections [16]. Most undocumented immigrant workers are employed in what Allan and Blustein term “precarious work” [17]. Having precarious work in early March 2020 significantly predicted job loss due to COVID-19 in May 2020; among those who remained employed, more precarious work predicted lower fulfillment of survival needs over time [17]. Job loss is a well-documented threat to mental and physical health [18,19]. Schaller and Stevens’ study of 10,000 individual job losses in the US found that job loss is associated with long-term increases in mortality rates and worsened mental health [20]. Job insecurity can also affect health. Several studies reported declining mental health in Latino communities during the pandemic, driven by job insecurity and job loss, misinformation, limited access to healthcare, and social isolation [21,22,23,24]. Job loss and changes were worse for undocumented and non-citizen Latino immigrants [25]. Fear of lost wages or job loss during the pandemic led many undocumented workers to continue working, even when sick, to afford food and housing [26,27]. At a federally qualified health center (FQHC) in Texas serving underinsured and uninsured children, more than 90% of Latino families surveyed (*n* = 200, of whom most were undocumented) reported food insecurity; among primarily Spanish-speaking families, 64% reported food insecurity worsening as a result of job loss due to the pandemic [28].

Even in the absence of a pandemic, Latino immigrants often receive the fewest workplace protections compared to other groups, and, thus, are the most marginalized of workers. More than 44% of U.S. noncitizens and 68% of undocumented immigrants are immigrants from Spanish-speaking countries [29,30]. There are 8.3 million undocumented Latino immigrants in the U.S., accounting for 16% of the Latino population. Compared to the general Latino population, Latino immigrants are more likely to live at or below the poverty threshold [31]. Poverty alone is associated with poorer access to healthcare, greater food insecurity, poorer air quality, increased exposure to violence, and lower-quality education, all of which contribute to worse health outcomes [32,33]. Furthermore, there are stark occupational, income, and racial disparities in access to and use of paid leave from work [34]. Many low-wage workers lack paid sick leave, and those without are more likely to go to work sick and to forgo medical care for themselves and their family members, problems highlighted by the COVID-19 pandemic [35].

While multiple studies highlight the importance of employment as a determinant of health for Latinos, the underlying pathways through which workplace conditions affect their health remain unexplored. Because Latino immigrants were highly vulnerable to job loss and work changes during the COVID-19 pandemic, especially in its most disruptive and economically turbulent first months, the theoretical framing of workplace dignity may be highly pertinent for understanding the relationship of work to health for Latino immigrants. This framing may also thus illuminate approaches to improving Latino immigrant health going forward.

### 1.2. Our Study

Our objective in this study was to explore the relationship between multiple dimensions of workplace dignity and the health of Latino immigrants during the early months of the COVID-19 pandemic. We draw on qualitative interviews with Latino immigrants who were clients of a federally qualified health center and living in an urban area with a large proportion of Latino immigrant residents. Our analysis revealed that the pandemic disruptions affected Latino immigrant dignity of and at work, including the inherent, earned, and remediated dignity that they derived (or were denied) from their work or unemployment. In turn, our investigation suggests that dignity of and at work affected these Latino immigrants’ health by worsening their mental health and exacerbating their pre-existing health conditions. We conclude by offering a new conceptual model for how Latino immigrant health is shaped by dignity of and at work.

## 2. Materials and Methods

### 2.1. Setting

Our study was conducted in the Washington, D.C., metropolitan (D.C. metro) area, which encompasses D.C., northern Virginia, and parts of Maryland, home to 425,000 undocumented immigrants [36]. Almost one million Latinos live in the D.C. metro area, comprising 15.3% of the area’s total population and making it the twelfth largest population of Latinos in the country; more than half of Latinos in the D.C. metro area (53.1%) are foreign-born [37]. Washington, D.C. is home to 12,000 undocumented Latino immigrants, and an additional 58,000 live in neighboring Prince George’s County, Maryland [38].

Among U.S. cities, the D.C. metro area has the third highest proportion of immigrants from Central America, especially from El Salvador [36]. The D.C. metro area has the highest concentration of El Salvadoran Temporary Protected Status (TPS) holders in the country [36]. TPS status protects immigrants from being detained, creates eligibility for a work permit, and allows immigrants to travel outside of the country with authorization, but it does not confer any eligibility for benefits or assistance.

Approximately 4.7% of D.C.’s labor force is undocumented, and six percent of Maryland’s workforce is undocumented [36]. Many of these undocumented workers work for cash as street vendors, domestic workers, and construction workers. These workers were not eligible for federal pandemic aid, unemployment benefits, or other federal social assistance programs.

### 2.2. Community Partner

We worked in collaboration with La Clínica del Pueblo (La Clínica), a health center serving low-income Latino immigrants in Washington, D.C., and Prince George’s County, Maryland. Since 2007, La Clínica has been designated a Federally Qualified Health Center (FQHC), one that must meet stringent federal requirements and provide services to all, regardless of ability to pay. FQHCs are the primary source of healthcare for undocumented immigrants, given their ineligibility for Medicaid or other health insurance programs. Of the 6686 adult clients of La Clínica served from 2016 to 2019, 91% were immigrants. About half (44%) of their adult clients had less than a high-school degree and reported incomes below the federal poverty level (52%). La Clínica offers culturally and linguistically competent clinical primary health services, as well as community-based health and education programs. All direct service staff are bilingual, and most are Latino immigrants themselves. La Clínica delivers their services through health promoters, trusted community members who educate their peers and facilitate access to social services such as legal aid, food security, and housing [39].

### 2.3. Recruitment

A health promoter approached La Clínica clients as potential participants for the study. Clients were asked if they were interested in conducting a phone or video conference interview about the social, economic, and health impacts of the coronavirus pandemic on Latino immigrants, as well as the resources that helped them to overcome pandemic-related challenges. Participants were eligible if they were a La Clínica client; resided in Washington, D.C., or Prince George’s County, Maryland; were aged 18 or older; were Latino; were an immigrant (foreign-born); and were Spanish- or English-speaking. Participants were diverse in the following characteristics: gender identity, sexual identity, pre-existing health conditions, experiences with domestic violence, experiences with HIV, and place of residence. People who expressed interest in participating were called by a prospective interviewer. The interviewer explained the study’s purpose and then reviewed eligibility criteria with the participant. Interviewers explained that the potential participant’s involvement in the study would not affect receipt of services from La Clínica. Health promoters shared the participants’ first names for confirmation purposes. Records of participants’ real names were then destroyed by the interviewers to ensure confidentiality. If the participant met the criteria and expressed consent to participate in the study, the interviewer scheduled a time for the interview. Prior to the interview, the participant received a confirmation call from the interviewer verifying the time and date of the interview. We stopped recruitment once saturation was reached with respect to key themes.

### 2.4. Data Collection

Interviewers were bilingual (English and Spanish) master’s students with extensive training in qualitative interviewing techniques. All interviews took place between June and December 2020 through a secure video conferencing platform. Informed consent was verbally obtained at the beginning of each interview, and the audio of each interview was recorded. Each interview lasted between 60 to 90 min. Upon completion of the interview, each participant was mailed a USD 60 gift card, and then the address record was destroyed.

The interview guide was semi-structured to encourage conversation between the interviewers and participants. Interview questions focused on the effects of COVID-19 on participants’ day-to-day lives, health (mental and physical), and employment. Participants were asked if/how their employment had changed since the onset of the pandemic, as well as how their day-to-day lives had changed due to stay-at-home orders. Participants were also asked to share any instances of social discrimination they had experienced during the pandemic and how they coped with it. At the end of the interview, participants were asked a multiple-choice question about their immigration status with the following response options: citizen/permanent resident for more than 5 years, permanent resident for less than 5 years, TPS or Deferred Action for Child Arrivals (DACA), undocumented, prefer not to respond, or other. Interviewers reminded participants that the information would not be shared with police or Immigration Customs Enforcement.

### 2.5. Ethics

All participants gave verbal informed consent in Spanish before completing the study interview. The study protocol was approved by the American University Institutional Review Board (IRB-2021-3).

### 2.6. Data Analysis

All audio recordings were transcribed in Spanish and imported into Dedoose, an online qualitative research software program, for coding and analysis. We used deductive reasoning, as we were guided by a general theory relating work to health. Data analysis consisted of the following steps. Six team members, including the Principal Investigator, were involved in the analyses. First, each team member read six unique transcripts to familiarize themselves with the content. The team then worked together to formulate a codebook. The first codes were created based on the interview guide questions. The next codes were created based on the new themes that arose from the transcripts. The team created definitions for each code to describe the code’s meaning.

To ensure coding consistency, all members of the team coded the first interview blindly; each team member was unaware of how the others were coding. The team then compared their coded transcripts. Places where team members diverged in their coding were discussed and consensus was reached as to how and when to apply a code. Following this process, the team refined the codebook, and each team member coded several interviews. The Principal Investigator reviewed all coded interviews for consistency. Each team member wrote memos during analysis. From these interpretations, the team identified dignity of work and dignity at work as themes. The participant quotes used in this paper have been translated into English.

## 3. Results

### 3.1. Participants

We interviewed 29 participants, most of whom were unemployed at the time of the interview (Table 1). The majority were between 26 and 49 years of age, a little more than half were female, and 6.9% were transgender women. The majority resided in D.C., and 37.9% resided in Prince George’s County, MD. Regarding immigration status, 28.5% of the participants reported being undocumented, 14.3% had a temporary status or work permit, and 25% were citizens or permanent residents. Notably, 17.9% identified as “other” documentation status and 10.7% preferred not to respond. El Salvador was the most common country of origin.

### 3.2. Theme 1: Dignity at Work

The theme of dignity at work was relevant to participants who were employed at the time of their interview, who made up one-third of the sample. These participants worked as construction workers, caretakers, restaurant employees, and cleaners during the first few months of the COVID-19 pandemic. We use pseudonyms to protect participants’ identities.

Several participants’ accounts suggested that COVID-19 had disrupted their inherent dignity, whether by forcing them to work at a distance from others or because of stigma associated with the virus itself. Participants who worked during the first months of the pandemic reported that social distancing measures in the workplace—working fewer hours and with fewer people—made them feel lonely and isolated. Maria, who often worked alone, said “*It’s really nice to have someone to talk to [the interviewer] because it doesn’t happen every day.*” (Undocumented, Female, 3 years in U.S.) Maria said that working alone compounded the social isolation she experienced at home.

A few participants reported being sick from COVID-19. They said they were stigmatized by fellow employees when they returned to work. Valeria, a U.S. permanent resident who lived in the US for over 20 years and worked as a cleaner, said she likely caught COVID-19 from a coworker whose brother was sick with the virus. Valeria said that her coworker did not disclose his COVID-19 exposure to the manager. Consequently, Valeria’s coworkers believed that she was the one who brought the virus into the workplace. When she returned to work after recovering, Valeria said: “*I felt ashamed. When I went back to work the other employees would stay away from me. I was discriminated against because they thought that I caught COVID outside of work.*” These interactions in which participants were stigmatized or isolated demonstrated loss of inherent dignity, or respectful interactions, at work.

Participants’ accounts also indicated that COVID-19 had highlighted their lack of earned dignity. Several participants said they took on extra burdens at work—like working flexible shifts and purchasing their own personal protective equipment (PPE)—that were not compensated by their employers, indicating a lack of earned dignity. Diana who worked in a daycare described some of the extra financial burdens: “*[My] clothes are discolored because they have to be put in hot water… I have to wash them every day. The masks are disposable and unsustainable. I cannot afford to buy a pack of disposable masks every day.*” *(Other immigration status, Female, 3 years in U.S.)* Another participant, Karen, shared that the time it took her to get to work was not worth the USD 30 she made per day. However, her employer would neither increase her pay nor give her longer shifts, so she quit her job.

When Valeria initially felt ill from COVID-19, her manager did not believe she was sick. He threateningly told her he needed to speak to her about missing work. Valeria explained how this situation made her feel: “*I am a person who is always at work. I am a responsible person in my work. We have people there that do not always come to work. And they don’t say anything to these people…*” Additionally, Valeria shared that she was not given severance pay when she had COVID-19 because this compensation was only provided to those who could prove they were infected at work. When a few of Valeria’s coworkers got sick immediately after her, they were fully compensated for their recovery time. The lack of compensation for sick days made Valeria feel that her contributions were unappreciated, a denial of earned dignity. Several participants related accounts that suggested they were denied remediated dignity. Some reported being forced to work multiple jobs due to pandemic-related workplace closures. For example, one participant, Fernando, whose manager promised him regular work, was told that his workplace would be immediately, permanently closed due to a lack of customers in the early months of the pandemic. Fernando described feeling “*disbelief*” that the employer would make a life-altering decision on such short notice: “*If my restaurant tells me that there’s no more work, then I’ll have no food to eat. We Latinos live day-to-day…we aren’t able to prepare for the future.*” *(Undocumented, Male, 7 years in U.S.)* Many participants echoed this sentiment, feeling that their employers were not aware of the hardships they faced. By failing to acknowledge these structural inequalities that placed extra stress on Latino immigrants during the pandemic, employers denied remediated dignity.

Some participants reported that their employers prioritized productivity over their physical well-being. For example, Gabriela who worked at a restaurant was asked by her manager to help wrap and pack gloves for Amazon. Gabriela described working every day until she was exhausted and had swollen hands. When Gabriela expressed her exhaustion to her manager, she was pressured to continue overworking, and she felt she could not quit because she needed the money to survive. Valeria’s manager similarly disregarded her health concerns: “*I got sick and my voice was completely gone. I called [my boss] to tell him I was sick. He was trying to force me to go to work. He was threatening me. What a thing, he wasn’t doing this with other [non-Latino] people.*” This discriminatory behavior indicated an absence of remediated dignity.

Table 2 summarizes the lack of inherent, earned and remediated dignity observed among the *employed* Latino immigrant participants in our study. However, it is important to note that those who were employed were in the minority among our participants.

### 3.3. Dignity of Work

All three types of dignity also applied to participants who lost their jobs or were underemployed during the first few months of the pandemic, who made up two-thirds of our sample. Job loss and unemployment were experienced as distressing and destabilizing. Several participants mentioned that they were the first to be fired because they were undocumented. When the pandemic struck, Julia and her husband lost all three of their jobs, and they worried about the effects of not being able to send money to children living in El Salvador. *(Undocumented, Female, 1 year in U.S.)*

A few participants shared that they had quit their jobs at the onset of the pandemic to avoid getting sick. Alejandra convinced her husband to stop working because his co-workers had all contracted COVID-19. Yesenia shared that she “*didn’t want to search [for jobs] anymore*” because it felt “*very dangerous*”. A few participants reported being underemployed, meaning that they had jobs but were working too few hours to support themselves. Underemployed participants almost exclusively described themselves as unemployed because of their lack of steady and sufficient income. Participants of all immigration statuses and genders experienced underemployment. As Miguel mentioned, “*It’s difficult because the capacity of the premises has really been reduced and the staff has too. I have some friends who only work three days, two days, or even just a few hours. We’re all looking for options to work because we need to cover our needs.*” *(Citizen/legal permanent resident, Male, 10 years in the U.S.)*

Several undocumented unemployed participants reported experiences that suggested a lack of inherent dignity: feeling abandoned, disrespected, and discriminated against because they did not qualify for government assistance during the pandemic. As Rosibel shared, “*During this time is when an immigrant is in most need, yet it was when we felt most discriminated against. They gave that $1200 to people who had a Social [Security number]. But what of those that don’t have anything and who are mothers or fathers with a family? Those are the things of the government I don’t understand. How is it that in reality there is so much inhumanity...so much injustice.*” (Undocumented, Female, 16 years in U.S.)

This sentiment was echoed by Yesenia: “*When the checks from the government were sent out, us undocumented did not receive that check... And so for me that is a form of discrimination.*” (Undocumented, Female, 16 years in U.S.) However, this feeling was not exclusive to the undocumented, as Juan, a participant with TPS, reported: “*...Up until now we have not received any other help...not even the check they gave out at the beginning, the one from the government. Because my wife doesn’t have a Social Security number, we couldn’t collect that [check]. It doesn’t seem fair. We are all here, we all work. In my case, I have 20 years of paying yearly taxes...and it does not seem right that they can’t give [the check] to you.*” *(TPS, Male, 20 years in U.S.)*

Albert, an undocumented participant, tearfully shared that the pandemic was difficult to deal with because he could only afford to live day-to-day: “*We were not prepared. In my mind, I never thought I would be without work for this long. I wasn’t ready to feed myself for three months and pay all the bills. I think that if I did not have my family, this would have been the end of me, because this is cruel [starts to cry].*” *(Undocumented, Male, 7 years in U.S.)*

Participants experienced the lack of government support as cruel and discriminatory, despite having worked for a long time and paid taxes in the U.S., representing a lack of inherent dignity towards these workers.

Participants who struggled with unemployment described how they felt a lack of earned dignity. Juan, who lost two jobs where he had worked for the past eight years, described feeling “*incapable*” due to lost work. Albert tearfully shared that his parents in El Salvador were sending money for his rent because he could only find work for two to five days per month, and his self-esteem had suffered as a result. He said his parents were encouraging him to return permanently to El Salvador.

Some participants described feeling forced to quit their jobs because of the circumstances posed by the pandemic. For example, some female participants left their jobs to provide care for their children. Gisell described having to leave her ideal job: “*Daycares were closed, schools were closed. Where does one take the children? Who takes care of them… I have no one to leave the children with.*” *(Citizen/Permanent Resident, Female, 19 years in U.S.)*

Another participant, Daniela, was forced to stay home with her children when the children’s father abandoned them. She also shared that she rented out her children’s room because she needed the money to pay rent. The inability of these participants to keep working prevented them from experiencing the earned dignity that comes from being employed.

Still other unemployed participants felt intentionally excluded from the rehiring process after the stay-at-home orders were lifted, essentially denying them remediated dignity. Many said that they were never contacted by their previous employers when businesses reopened, despite being told they would be. A few of these participants expressed that waiting to hear back from their previous employers prevented them from moving forward with their job searches.

Both transgender participants were fired from their jobs and were never rehired, despite being told they would be by their former employers. Sara was fired from a shop where she had worked for 30 years. She was told she would be called back to work when the shop reopened, but that did not happen: “*It was unfair...I was one of the older people… They didn’t even expect me to retire. [They said] well, we don’t need you anymore.”* Sara also shared that her employer offered her job to a younger worker: *“I’m not too innocent to understand things like that…It hurt me a lot. I spent my whole life in that job.*” (*Citizen/Permanent Resident, Transgender Female, 30 years in U.S.)*

Another transgender participant, Valentina, was fired from a restaurant she had worked at for 16 years. Two months later, the restaurant reopened, and Valentina learned that others were rehired. By purposefully excluding these transgender women from the rehiring process, employers denied them remediated dignity.

Table 2 summarizes the lack of inherent, earned and remediated dignity observed among *unemployed* Latino immigrant participants in our study.

### 3.4. Effects of Dignity on Health

Regardless of their employment status, participants expressed that their working conditions or lack of employment during the early months of the pandemic had a negative effect on their physical and mental health. Employed participants shared that the lack of dignity they experienced at the workplace deteriorated their mental well-being. Employers’ disregard of the structural hardships they were facing and extra precautions they had to take made the workplace a site of long-term anxiety for many participants. A few participants who were employed were infected with COVID-19. Almost all of them believed they were exposed to the virus at work, confirming their fears. In Valeria’s case, working during the pandemic exposed her and her loved ones to the COVID-19 virus. Among unemployed participants like Miguel, Juan, Gisell, and Daniela, the stress of experiencing financial precarity took a toll on their physical and mental wellbeing. They described experiencing weight gain, substance abuse, and depression and anxiety. Participants described how unemployment worsened their pre-existing health conditions. As Yesenia shared, “*My illness [uterine fibroids] developed and then this (unemployment) situation...My anxiety has been brewing.*” Before COVID-19, Yesenia lived a very active lifestyle as a fryer at a restaurant. After she lost her job, she started feeling “*stuck*” with herself: “*This pandemic has affected me quite emotionally. It has worsened my mood a lot.*”

Another participant, Mateo, was recovering from alcoholism at the onset of the pandemic. Three months after losing his job, he began to run out of savings: “*I felt very stressed. I made the bad decision to drink again, and I was arrested… for a DUI.*” *(Undocumented, Male, 8 years in U.S.)*

In another case, Sara, a transgender participant who was living with HIV, learned at a routine blood test that her sugar and blood pressure levels were rising. She said: “*If I were working, it wouldn’t be like this… My work has always been physical because I have been a laborer in every position… I have gained weight because… at home you only eat.*”

Other participants relayed how their experiences with unemployment had contributed to anxiety and depression. Ximena, a woman who had been living with TPS for 16 years in the U.S., described how pandemic-related job loss brought about depression: “*Before, I could be distracted at work. Now, I am isolated from people. I can’t do it anymore.*” Mateo shared that it had been months since he lost his job: “*I am still unemployed. I still have to spend so much time in my house. My health has deteriorated.*” *(Undocumented, Male, 8 years in U.S.)* Even employed participants like Valeria, Fernando, and Gabriela also reported worsening mental health due to stressful conditions and a lack of dignity at work. Some described intense fear of contracting COVID-19 at the workplace and anxiety about spreading it to their loved ones.

Both employed and unemployed participants reported experiences consistent with a lack of inherent, earned, and remediated dignity of work and at work, and they attributed worsening pre-existing health conditions and increased levels of anxiety and depression to these negative experiences.

## 4. Discussion

### 4.1. Denial of Workplace Dignity Leads to Adverse Mental and Physical Health Outcomes

Early in the COVID-19 pandemic, Latino immigrants in the D.C. metro area, already at elevated risk of acquiring COVID-19, faced a rapidly changing employment landscape that largely denied them inherent, earned, and remediated work-related dignity. Inequities deriving from structural racism were brought into stark relief by the COVID-19 pandemic, forcing an acknowledgment of the intersections between essential worker status, race, and health, as well as the centrality of how work—and dignity *of* work and *at* work—affects health. Our study is novel in its use of workplace dignity theory to describe Latino immigrants’ experiences during the pandemic and suggests how workplace dignity may have affected their health. Participants’ accounts link denial of dignity—through job loss, underemployment, poor/unsafe working conditions, lower self-esteem, and lack of paid sick leave—to their adverse health outcomes, including increased anxiety, poor metabolic control, substance abuse, and depression.

Existing literature on work and health does not sufficiently address the concept and importance of dignity of and at work and the health consequences of being denied inherent, earned, and/or remediated dignity. The literature has described how workplace discrimination is associated with increased workplace injuries among the Latino immigrant workforce [40,41,42,43,44], as well as adverse mental health effects, particularly among undocumented workers [45,46]. The higher rates of COVID-19 exposure, morbidity, and mortality during the early months of the COVID-19 pandemic, when many Latino workers were forced to work while the majority of Americans sheltered in place, are rightly conceptualized as workplace hazards that denied dignity to these essential workers [47]. Our study adds to a critically needed growing body of literature describing how quality of work, treatment in the workplace, and feelings about work affect workers’ mental and physical health [8,9].

### 4.2. Expanding the Paradigm on Work and Health to Include Workplace Dignity

Lucas’ dignity framework usefully expands our understanding of pathways through which job changes and job loss can affect health. While not originally intended to apply to health, being denied inherent, earned, or remediated dignity at work likely elicits a stress response and adds to immigrants’ allostatic load, a plausible causal pathway for health problems including sleep disturbances, cardiovascular disease, obesity, and diabetes [48,49,50]. Our participants faced an impossible quandary: work did not provide dignity and put them in danger of acquiring COVID-19; however, losing their job also had negative health impacts. While the plight of immigrant workers in the U.S. has received academic attention in recent years, highlighting issues of harassment, discrimination, wage theft, and unfair criminalization [51], as well as health inequities rooted in immigration enforcement policies [52], the direct and indirect impact of these injustices on workers’ physical and mental health has been underexplored. Further research is needed to assess the short- and long-term health outcomes of denial of workplace dignity, including discrimination, job loss, underemployment, and unemployment on immigrant workers.

### 4.3. A New Conceptual Model on Workplace Dignity and Health for Immigrants

**Figure 2:**
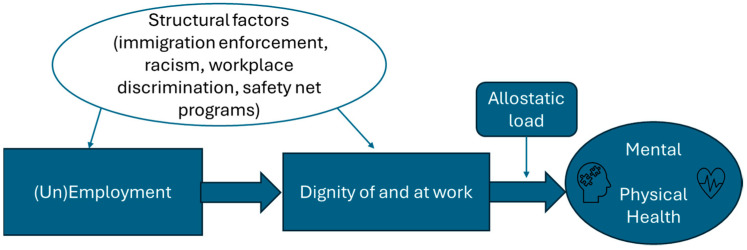
Proposed conceptual model linking workplace dignity to health.

Drawing on our data and Lucas’ theory of dignity, we present a conceptual model for how future research could investigate the relationship between dignity and health for immigrants (Figure 2). While Lucas’ framework is useful for exploring the different manifestations through which workers can experience or be denied dignity at work, it does not adequately account for structural inequities, such as federal or state policies, that can constrain or deny both employment and dignity. While remediated dignity acknowledges that workplace inequalities exist and can contribute to denial of dignity, this concept insufficiently applies to those who lack legal rights as workers, like undocumented immigrants, and does not adequately incorporate structural sources of racism and inequality.

Lucas’ model could thus be helpfully expanded to include structural determinants of attainment of dignity at work. As a positive example, the presence of paid leave policies in some states, a structural determinant, conferred remediated dignity for workers and commensurately higher COVID-19 vaccination rates in those states [53]. On the other hand, Latino essential workers have lower access to health insurance coverage [54] and paid leave, structural factors which deny remediated dignity at work and put them at greater risk of acquiring COVID-19 during the pandemic [4,16,55,56].

Structural racism constrains opportunities for Latino immigrants and limits dignity of and at work [1,43,57]. Rathod notes that immigrants’ occupational risks are shaped by very different structural forces than those of nonimmigrants, with multiple legal regimes intersecting to shape these risks [43]. Similarly, workplace dignity is an as-yet-unrecognized social determinant of health that can usefully inform social epidemiology inquiries. For example, regardless of their legal status, Latino immigrants who live in environments where they have greater fear of deportation, or where they fear that accessing programs may lead to deportation, inhibiting what Rathod terms “safety-related assertion of rights” [43,58,59,60], are likely to avoid accessing healthcare [61]. For Latino essential workers who worked during the pandemic, their elevated exposure to COVID-19 because of lack of dignity at work, along with their overall poorer health [62] associated with underlying poverty [63], resulted in higher COVID-19 death rates for Latino essential workers compared to white essential workers [64,65]. Additional research is needed to plumb the intersections between work, dignity, and health, particularly for workers with precarious employment.

Being undocumented in the U.S. is linked to structural vulnerabilities including workplace discrimination, as well as workplace stressors, and has been associated with increased anxiety and depression symptoms among Latinos, as well as increased risk of injury at work [40,42,45,66]. Undocumented workers were often first to be fired during the pandemic, with no recourse. Documentation status was also a clear barrier to federal relief programs [57]. Structural vulnerabilities associated with immigration status have been largely overlooked in conceptual models and constructs of worker health, even though undocumented workers generally have more stressful life experiences than documented workers and have a higher risk of exposure to occupational hazards and dangerous worksite conditions [44,46]. The COVID-19 pandemic laid bare troubling intersections between essential worker status, immigration status, race, and health outcomes. Conceptualizations of workplace dignity could benefit from what Kaufmann et al. term a “negative approach to human dignity” that acknowledges the damage to immigrant workers—in the form of ridicule, humiliation, and dehumanization—that results from denial of workplace dignity perpetrated by employers as well as policies that fail to safeguard these groups [67].

### 4.4. Policy Implications

Critically, a policy response is needed to address immigrant health, particularly for those immigrants who lack legal employment protections. During the early months of the COVID-19 pandemic, the U.S. government took few actions to protect its immigrant essential workers; respondents in our study shared experiences of working multiple jobs and being forced to work out of financial desperation. Designating Latino workers as “essential” yet omitting them from policies to support worker safety workplace protections—an experience Rubenstein et al. documented among Latino meatpacking workers early in the pandemic—reflects gross structural discrimination that cost both livelihoods and lives [68]. Moreover, the U.S. Supreme Court struck down the Occupational Safety and Health Administration (OSHA) mandate that large employers require workers to be either vaccinated or fully masked at work, a decision that ultimately prolonged the pandemic, exposed more essential and immigrant workers to COVID-19, and extended economy-disrupting labor shortages [69]. Normalizing injustices suffered by groups denied basic social and economic security is at odds with both modern democracy and humanism.

Policy responses to public health and economic emergencies should reflect a commitment to inclusion to protect vulnerable groups from inequitable treatment [70]. Pandemic relief programs included stimulus payments, expanded Child Tax Credits, and continuous enrollment in Medicaid, with a growing research base finding large benefits for recipients [71,72,73]; however, none of these programs were available to undocumented populations. While many of these policies were enacted during the COVID-19 pandemic for low-income people, they should be extended to Latino immigrants regardless of documentation status [74,75,76]. In addition to policy responses during public emergencies, policy responses such as basic workplace safeguards like paid sick and family leave policies, social protection programs that address housing and food security, and health insurance for all workers would help protect vulnerable groups and the general population alike [77]. A growing number of states and localities are enacting paid leave mandates (e.g., Washington State) and extending public health insurance access to undocumented immigrants (e.g., Washington, D.C.), and rigorous research is needed to understand the effects of these policies in narrowing health and other disparities the aftermath of the pandemic [74,75,76].

### 4.5. Limitations

Our study population was broadly reflective of Latino immigrants nationally: early in the pandemic, most either lost their jobs or had to work while more privileged workers sheltered in place. However, given the way participants were recruited through an FQHC, we should note that our sample may be among the more “advantaged” undocumented Latino immigrants who were accessing and receiving healthcare. Other Latino immigrants may be disconnected from the health system, likely resulting in health outcomes worse than our sample. Additionally, the qualitative nature of this study lends itself to self-selection bias; La Clínica patients who were contacted about this study may have had specific viewpoints, experiences, fears, or interests that influenced their decision to participate. Nevertheless, we stopped interviewing when we reached saturation, suggesting that the themes presented here were relevant for many people in our study population. While our study design allows us to construct a portrait of the range of Latino immigrant experiences with precarious employment and unemployment, dignity of and at work, and physical and mental health in the early days of the pandemic, we cannot compare participants’ experiences to before the pandemic or with those of non-Latino immigrants in the metro D.C. area. Data were collected from June to December 2020 when job losses were steep and primarily affected sectors that disproportionately employ Latinos. Ideally, we would have followed participants before and throughout the pandemic to learn whether these job losses were temporary or had lasting impact on their physical and mental health. Future studies could explore the relationship between workplace dignity and health during non-pandemic times, and whether improvements in workplace dignity can mitigate or reverse poor health conditions.

## 5. Conclusions

We presented a conceptual model of the relationship between workplace dignity and health that considers structural aspects affecting dignity and health for Latino immigrant workers. The dignity of Latino immigrant workers in the US, particularly undocumented workers, is generally overlooked by both employers and policymakers; these trends became apparent and had cascading adverse health effects during the COVID-19 public health emergency. Denial of dignity compounded the elevated risk of infection with COVID-19 that many Latino immigrants faced as a function of their social and economic positioning and employment conditions. Research, as well as social, legal, and policy protections, are needed for undocumented Latino immigrants who are at greatest risk of harm due to denial of dignity of and at work.

## Figures and Tables

**Table 1:** Demographic characteristics of participants (n = 29).

Characteristic	Categories	Number of Participants	% (n)
Age (n = 29)	18–25 years	4	13.8%
	26–49 years	18	62.1%
	50+ years	7	24.1%
Gender identity (n = 29)	Cisgender women	15	51.7%
	Cisgender men	12	41.4%
	Transgender (male to female)	2	6.9%
Employment status during COVID-19 (n = 28)	Employed	9	32.1%
	Unemployed	19	67.9%
Place of residence (n = 29)	Washington, D.C.	18	62.1%
	Prince George’s County, MD	11	37.9%
Immigration status (n = 28)	Undocumented	8	28.5%
	TPS	4	14.3%
	Permanent resident/citizen (≥5 years)	7	25%
	Permanent resident/citizen (<5 years)	1	3.6%
	Other	5	17.9%
	Prefers not to respond	3	10.7%
Country of origin (n = 26)	El Salvador	14	53.9%
	Guatemala	6	23.1%
	Mexico	2	7.7%
	Honduras	2	7.7%
	Dominican Republic	1	3.8%
	Venezuela	1	3.8%

**Table 2:** Summary of Latino immigrants’ experiences of the lack of three types of dignity at and of work during the early months of the COVID-19 pandemic.

Dignity Type	Lack of Dignity *at* Work	Lack of Dignity *of* Work
**Inherent**	social isolation; COVID-19 stigma at work	lack of government assistance perceived as lack of respect for immigrants who pay taxes
**Earned**	extra burdens at work, including COVID-19 exposures, that were not acknowledged by employers; lack of compensation for sick leave	feeling like their workplace contributions were not acknowledged; lack of self-esteem from not working; forced to quit their jobs because of lack of workplace protections
**Remediated**	stressful and intimidating work conditions; losing work without notice	excluded from the rehiring process

## Data Availability

The original contributions presented in the study are included in the article; further inquiries can be directed to the corresponding author(s).
